# Persistent Organic Pollutants and Obesity-Related Metabolic Dysfunction: Focusing on Type 2 Diabetes

**DOI:** 10.4178/epih/e2012002

**Published:** 2012-01-27

**Authors:** Duk-Hee Lee

**Affiliations:** Department of Preventive Medicine, Kyungpook National University School of Medicine, Daegu, Korea.

**Keywords:** Diabetes, Obesity, Organochlorine pesticides, Persistent organic pollutants, Polychlorinated biphenyl

## Abstract

Even though obesity is a well-established risk factor of type 2 diabetes, there is emerging evidence that persistent organic pollutants (POPs), a variety of lipophilic chemicals accumulated in adipose tissue, may be critically involved in the pathogenesis of type 2 diabetes. Among various POPs, serum concentrations of organochlorine pesticides and polychlorinated biphenyls (PCBs) were the most strongly and consistently linked to type 2 diabetes in both cross-sectional and prospective studies. In particular, obesity did not seem to be related to type 2 diabetes among persons with very low serum concentrations of POPs, suggesting a more fundamental role of chlorinated POPs in the pathogenesis of type 2 diabetes. These POPs were also associated with obesity, insulin resistance, and adverse lipid profiles like high triglyceride and low high-density lipoprotein (HDL) cholesterol among persons without diabetes, all metabolic dysfunctions commonly observed before developing type 2 diabetes. Recent animal studies supported the findings from epidemiological studies. If all these findings on POPs are true, it suggests that any effort to reduce the external and internal exposure to POPs would be necessary to decrease the social burden of type 2 diabetes.

Type 2 diabetes is one of the most common chronic diseases in both developed and developing countries and continues to increase in numbers and significance as economic development and urbanization lead to changing lifestyles. Type 2 diabetes is usually associated with obesity, physical inactivity, older age, or family history of diabetes. Among known risk factors, obesity is the most important and well-established one. However, there is emerging evidence that persistent organic pollutants (POPs) accumulated in adipose tissue, rather than obesity itself, may be critically involved in the pathogenesis of type 2 diabetes.

POPs are a group of chemicals with common properties such as lipophilicity and persistence. The examples of POPs are polychlorinated dibenzo-p-dioxins, polychlorinated dibenzofurans, polychlorinated biphenyls (PCBs), and organochlorine (OC) pesticides. Although most chlorinated POPs such as PCBs and OC pesticides were banned several decades ago and dioxins are strictly regulated in most developed countries, the general population is currently exposed to these chemicals through food consumption because they have widely contaminated our food chain, especially fatty animal food. In addition, POPs which were accumulated in adipose tissue from previous high exposure are a source of internal exposure as they keep being release from adipose tissue to circulation and reach critical organs.

In a cross-sectional study among the U.S. general population published in 2006, serum concentrations of chlorinated POPs were strongly associated with the prevalence of type 2 diabetes [1]. After adjusting for established risk factors of type 2 diabetes, the prevalence of type 2 diabetes was 15 to 40 times higher among subjects with detectable concentrations of chlorinated POPs compared to those with very low concentrations of chlorinated POPs. In addition, the associations became stronger as people were obese. Although chlorinated POPs with dioxin properties have been known as the most toxic chemical, OC pesticides and PCB congeners, rather than dioxins, were more strongly associated with type 2 diabetes. Particularly, it was unexpected that obesity was not related to type 2 diabetes among persons with very low concentrations of POPs and diabetes itself was rare even among the obese, suggesting a more fundamental role of chlorinated POPs in the pathogenesis of type 2 diabetes [2]. In following studies, serum concentrations of these POPs were also associated with insulin resistance and adverse lipid profiles like high triglyceride and low high-density lipoprotein cholesterol among persons without diabetes, which are all metabolic dysfunctions commonly observed before developing type 2 diabetes [3,4].

However, these findings seemed to be puzzling because the body burden of chlorinated POPs has declined worldwide over the recent several decades after banning of production and usage in most developed countries while type 2 diabetes show a recent worldwide epidemic. If chlorinated POPs are really important in the development of type 2 diabetes, how is this kind of mismatch of time trend possible? Our explanation on this issue was that a low dose but persistent exposure might be more harmful than high dose exposures if chlorinated POPs are involved in the pathogenesis of type 2 diabetes as endocrine disruptors.

Larger effects from low dose exposure than high dose exposure have been proposed as possible biological responses of chemicals with endocrine disrupting properties [5]. It is different from traditional toxicology, which assumes a linear dose-response relation. In fact, POPs are well-known endocrine disruptors. In biological systems, there is a linear dose-response relation only up to a dose that occupies about 10% of receptors. As doses of hormone further increases, the occupancy rate does not linearly increase. In addition, a linearity of biological response is observed at much lower doses of hormones than that showing linearity with receptor occupancy. Under the high dose exposure of hormones, there is even down-regulation of receptors as the dose further increases [6]. Thus, chemicals with endocrine disrupting properties can make inverted U dose-response curves with certain biological endpoints.

These cross-sectional associations were recently confirmed by several prospective studies, even though the kinds of individual POPs predicting type 2 diabetes and the shapes of the dose-response curves were not exactly the same among the studies [7-10]. Also, some OC pesticides and PCBs predicted insulin resistance and dyslipidemia among persons without type 2 diabetes [8]. One recent animal study strongly supported the findings from epidemiological studies. Rats exposed to POPs through the consumption of fish oil contaminated with various POPs developed dyslipidemia, hepatosteatosis, and insulin resistance [11]. Also, POPs down-regulated insulin-induced gene-1 and Lpin1, two master regulators of lipid homeostasis [11].

Importantly, the prospective studies among young adults showed clear inverted U-shaped associations [8,9]. In the situation of inverted U-shaped associations, it is crucial to note that the dose-response curve in human studies can vary depending on the distribution of chemicals of a specific population. The whole range of inverted U-shaped associations can be observed only when the study population has a wide range of concentrations of the chemical ranging from very low doses to very high doses. Usually, it is well known that the extent of contamination of a specific chemical greatly varies among different countries and different time periods or different sites of one country.

Another interesting finding was that some POPs even predicted future obesity [8]. Consistent with the human finding, the animal study also showed that rats exposed to POPs clearly developed visceral obesity [11]. At present, the worldwide epidemic of obesity is attributed to both a high calorie diet and a sedentary lifestyle. However, some of the numerous environmental pollutants that cause disturbance of endogenous hormonal regulation involved in weight homeostasis have been recently proposed as possible risk factors for obesity, referred to as environmental obesogens [12]. Although current available evidence has been mostly based on animal experiments focusing on effects of fetal exposure to chemicals, we expect more human evidence in the near future.

In conclusion, the results from a series of recent studies strongly suggest that environmental exposure to some chlorinated POPs may be involved in the increased risk of type 2 diabetes, as well as obesity and other obesity-related metabolic disturbances. If all these findings on POPs are true, it means that any effort to reduce the external and internal exposure to POPs would be necessary to decrease the social burden of type 2 diabetes.
